# The “Vulnerable” in the G7’s Vision: Children With Medical Complexities and the Call for Specific Strategies

**DOI:** 10.7759/cureus.76765

**Published:** 2025-01-01

**Authors:** Yudai Kaneda, Hirotomo Miyatake, Akihiko Ozaki

**Affiliations:** 1 Epidemiology and Public Health, School of Medicine, Hokkaido University, Hokkaido, JPN; 2 Pediatrics, Home-Care Clinic, Orange Medical &amp; Social Services, Fukui, JPN; 3 Department of Breast Surgery, Jyoban Hospital, Tokiwa Foundation, Fukushima, JPN

**Keywords:** children with medical complexities (cmcs), g7 hiroshima summit, inclusive society, japan, vulnerable people

## Abstract

The G7 Hiroshima Summit (May 19-21, 2023) established the "G7 Hiroshima Vision for Fair Access to Medical Countermeasures (MCMs)", which includes vaccines, drugs, and antidotes for chemical, biological, radiological, and nuclear emergencies. The Summit also led to the creation of the "Medical Countermeasures Delivery Partnership." This initiative aims to ensure equitable access to MCMs, emphasizing principles like equity, inclusivity, efficiency, affordability, quality, accountability, agility, and speed. Japan's extensive experience positions it to lead globally in developing support systems for vulnerable populations, particularly children with medical complexities (CMCs). Despite this, the Summit's discussions lacked specificity in addressing these children's unique needs. Advances in medical technology have increased the number of CMCs, highlighting the importance of home-based advanced care. Our experience shows that CMCs can participate in activities such as excursions and hikes with proper preparations, including adequate oxygen and electricity and broader societal support. Recognizing CMCs as unique community members is crucial for promoting social justice, human rights, and societal innovation. The G7 communique's vague reference to 'vulnerable people' underscores the need for more precise and individualized approaches.

## Editorial

The G7 Hiroshima Summit, conducted from May 19-21, 2023, addressed various issues, including support for Ukraine, nuclear disarmament, and infrastructure investment. A key topic was medical countermeasures (MCMs), that is, vaccines, antiviral drugs, antibiotics, and antitoxins designed to protect public health during chemical, biological, radiological, and nuclear emergencies. The outcome was the declaration of the "G7 Hiroshima Vision for Fair Access to Medical Countermeasures (MCMs)," leading to the inauguration of the "Medical Countermeasures Delivery Partnership." This initiative was designed to facilitate more equitable access to and delivery of MCMs, with guiding principles of equity, inclusivity, efficiency, affordability, quality, accountability, agility, and speed [1]. In this context, Japan holds the potential to emerge as a global leader in offering assistance to vulnerable populations such as children with medical complexities (CMCs), courtesy of its extensive experience and proficiency in this domain. However, the specificity of the relevant discussions seems inadequate in this summit.

Indeed, advances in medical technology have led to an increase in the number of CMCs who require support in their daily lives, elevating the importance of advanced care at home worldwide. In Japan, the number of CMCs using medical devices stood at 19,712 in 2018, approximately twice the number recorded in 2008 [2]. While the International Classification of Functioning, Disability, and Health (ICF) model promotes enabling children with disabilities to actively participate in society without waiting for functional improvement, the current system often makes it difficult for CMCs to venture outside as it necessitates additional caregivers, and does not always ensure accessible transportation options [3]. However, from our practice to date, we have found that even for CMCs, they can participate in activities such as excursions or hikes, provided the individual preparations necessary are made, for instance, securing sufficient oxygen and electricity, and broader societal support is available for their experiences [4-6]. 

This suggests that they are vital members of society who can grow both mentally and physically, and we must prepare all possible measures to support and foster their growth. Therefore, enabling CMCs will serve as a cornerstone for creating a more inclusive and equitable society that benefits both CMCs and other vulnerable populations. Moreover, developing ways to achieve this will also advance society as a whole by encouraging innovation in areas such as infrastructure and healthcare delivery [7].

Therefore, although the requisite assistance ought to vary according to each individual, necessitating a more nuanced and detailed assessment of both environmental and personal factors pivotal for societal participation, the recent G7 communique has, regrettably, defaulted to the generalized and simple terminology of "vulnerable people" [1]. This challenge cannot be addressed through a top-down approach alone. While the term "vulnerable population" encompasses diverse groups, even among CMCs, access to MCMs varies significantly depending on individual patient circumstances and available support systems. This underscores the importance of developing solutions that incorporate frontline experience and knowledge alongside policy initiatives.
